# Changes in Japanese Pharmacists’ Recognition of Their Role in Community Public Health before and after the Spread of COVID-19

**DOI:** 10.3390/pharmacy9030154

**Published:** 2021-09-09

**Authors:** Kayoko Takeda Mamiya, Akari Yoshida

**Affiliations:** Department of Pharmaceutical Education, Faculty of Pharmaceutical Sciences, Hokkaido University of Science, Sapporo 006-8585, Japan; 117A011@stu.hus.ac.jp

**Keywords:** community, COVID-19, public health

## Abstract

Background: In our previous study regarding infection prevention after COVID-19, many Japanese citizen respondents had not received education/training on infection prevention. However, a total of 47.7% (*n* = 143) of these respondents wanted to receive education from healthcare professionals regarding the methods and effects of infection prevention. Therefore, changes in recognition of the roles of Japanese pharmacists before and after COVID-19 were investigated. Methods: We conducted a survey to determine whether recognition of Japanese pharmacists’ roles, especially their role in public health in the community, changed after COVID-19. Results: A total of 93.9% (*n* = 307) of the pharmacist respondents showed an increased awareness of infection prevention. Before COVID-19, the hospital pharmacists (67.2%; *n* = 80) were more aware of infection prevention than were pharmacy pharmacists (51.7%; *n* = 74) and drugstore pharmacists (47.7%; *n* = 31). The number of pharmacists who felt that the role of pharmacists in the community had changed after the pandemic increased, but the numbers of community pharmacy pharmacists (51.8%; *n* = 74) and drugstore pharmacists (55.4%; *n* = 36) were found to be slightly higher than those of hospital pharmacists (47.9%; *n* = 57). Conclusions: In a society in which swift responses and changes are required, for individuals to work as medical personnel their ability to respond while always being aware of the needs of society is required now more than ever.

## 1. Introduction

Background: The COVID-19 pandemic that began in 2019 has forced people worldwide to change their lifestyles and has raised awareness about infection prevention measures among the public, including healthcare professionals. Given that some infection prevention measures, such as mask wearing, hand washing, hand disinfection, and social distancing, have become common worldwide, and that infection prevention measures as a whole do not greatly vary among countries, the differences in infection prevention measures have not garnered much attention [1,2]. Regarding infection prevention measures in Japan, the Ministry of Health, Labour and Welfare recommends social distancing (avoiding the ‘3 Cs’: closed spaces with poor ventilation, crowded areas and close contact), mask wearing and hand washing and disinfection with alcohol [3]. It is also possible that implementation awareness and the actual implementation of these measures have been increasing, given their widespread coverage by the media on a daily basis. However, mask wearing, methods of hand washing and disinfection can differ greatly among individuals. If this is the case, then there is a risk that effective infection control measures are not in place. However, although numerous studies have been published on changes in rates of sterilisation and the effects of conducting surveys and training for medical professionals on hand disinfection [4,5,6,7,8,9,10,11,12], few have targeted citizens. A survey on hand hygiene among pharmacists (who are also considered medical professionals) found that the rate of correct answers regarding situations in which hand hygiene and procedures for the use of fast-drying hand disinfectants are required increased after training sessions were held [11]. Thus, even healthcare professionals can have incorrect or insufficient knowledge of hand hygiene. This suggests that citizens could also have incorrect perceptions regarding this issue. Therefore, in a previous study, we conducted a survey of changes in Japanese individuals’ awareness regarding infection prevention before and after the spread of COVID-19 [13]. A total of 89.7% of the respondents (269 of 300 citizens) stated that their infection prevention awareness increased due to the spread of COVID-19, and the prevalence of those who took daily infection prevention measures increased after the spread of the virus. Most respondents could not answer correctly regarding infection prevention for the ‘parts of the hand disinfected’, ‘parts of the hand washed’ or ‘duration of hand washing’. These results may indicate that healthcare professionals or community pharmacists are unaware of the necessity of patient education with respect to infection prevention, as healthcare professionals consider that infection can be prevented by engaging in the commonly followed habits of Japanese individuals [13]. In fact, the responses indicating that at least 70% of the respondents had not received education or training on infection prevention (214 respondents had not received education/training on hand washing, and 256 respondents had not received education/training on hand disinfection), demonstrating that infections could be prevented by means of habitual behaviour by Japanese individuals. However, a total of 47.7% (*n* = 143) of the respondents wanted to receive education/training regarding the methods and effects of infection prevention from healthcare professionals [13]. Of these, only 21.7% (*n* = 31) wanted to receive education/training from pharmacists.

Purpose of this study: These findings could be due to the public being unaware of the role played by pharmacists in public health [13]. Moreover, while a review of the role of pharmacists during COVID-19 has been published, and while the International Pharmaceutical Federation (FIP) and World Health Organization provide information on COVID-19 in various countries on their homepages, none of the previous studies has included Japan [1,14,15]. In this study, we conducted a survey to determine the level of recognition of the role of Japanese pharmacists, especially their role in public health in the Japanese community, after the spread of COVID-19, and we made comparisons among community pharmacy pharmacists (who mainly deal with prescriptions for outpatients in hospitals), drugstore pharmacists (who mainly deal with the over-the-counter drugs) and hospital pharmacists.

Significance of this study: While the number of people with COVID-19 infections, the countermeasures taken, and the role of pharmacists differ across countries, a change in the consciousness of Japanese pharmacists is meaningful not only in individual cases but also for Japan as a whole. It is important for professional education to clarify the changes in the role of pharmacists required by citizens and to reflect these changes in education, as the FIP’s quality assurance has indicated [16]. Therefore, understanding pharmacists’ recognition of their roles based on citizens’ needs will be the first step in reviewing undergraduate and postgraduate education.

## 2. Materials and Methods

Research design: This was a survey study, and the target population was Japanese pharmacists, i.e., community pharmacy pharmacists, drug store pharmacists (who mainly deal with over-the-counter drugs) and hospital pharmacists. The participants were administered a questionnaire with 14 items related to infection prevention. The respondents were required to understand the content of the questionnaire, and only those who provided consent advanced through the screening process.

Recruitment and participants: From 20 May to 25 June 2021, a web-based survey was provided to pharmacist members registered through NEXTIT Inc. (http://www.nextit.co.jp/ (accessed on 8 September 2021)) in Kobe. The survey was provided only to those who understood and consented to the purpose of the present study.

Data analysis: Chi-square tests were used for analysis (differences among community pharmacy pharmacists, drugstore pharmacists and hospital pharmacists), and were conducted using SPSS Statistics 25.0 (IBM, Chicago, IL, USA). All chi-square tests were two-tailed tests. In addition, for Likert scale questions, the Kruskal-Wallis test was conducted after checking normality using the Kolmogorov-Smirnov test, with differences reported as significant if *p* < 0.05.

Ethical considerations: This study was approved by the Institutional Review Board of the Faculty of Pharmacy, Hokkaido University of Science (IRB approval no.: 20–06–017; date: 24 February 2021).

Protection of personal information. Given that this study was anonymous, the data processed from the web survey did not contain any information containing personal data, and individuals could not be identified. For the data file, however, a code was established so that only study personnel could access the data via a specific personal computer.

## 3. Results

### 3.1. Recognition of Infection Prevention before and after the Spread of COVID-19

The total sample consisted of 327 pharmacists including 143 (43.7%) community pharmacy pharmacists, 65 (19.9%) drug store pharmacists and 119 (36.4%) hospital pharmacists). The age distribution of the respondents was as follows: eight (2.4%) aged 20–29 years, 73 (22.3%) aged 30–39 years, 121 (37.0%) aged 40–49 years, 89 (27.2%) aged 50–59 years and 36 (11.0%) aged ≥ 60 years. Table 1 shows the characteristics of the respondents.

A total of 307 (93.9%) pharmacists stated that their “infection prevention recognition increased” due to the spread of COVID-19 (Table 1). The rate of 80 (67.2%) hospital pharmacists’ recognition (67.2%) of their role in infection prevention education for citizens or patients was significantly higher than that of community pharmacy pharmacists 74 (51.7%) or drugstore pharmacists 31 (47.7%) before the spread of COVID-19 (*p <* 0.05, chi-square test) (Table 2). The rate of hospital pharmacists’ recognition 103 (86.6%) of their role in infection prevention education for citizens or patients was significantly lower than that of community pharmacy pharmacists 133 (94.4%) or drugstore pharmacists 63 (96.9%) after the spread of COVID-19 (*p <* 0.05, chi-square test) (Table 2). Moreover, the rate of all pharmacists’ recognition of their role in infection prevention education remarkably increased after the spread of COVID-19 (Table 2). In addition, the rate of the conduct of citizen or patient education was also remarkably increased by pharmacists after the spread of COVID-19, especially regarding hand disinfection with alcohol, disinfection of each infectious disease and explanation of the infection route (Table 3).

### 3.2. Changing Role of Pharmacists before and after the Spread of COVID-19

To grasp the changing role of pharmacists in the community, we asked all the respondents “Do you feel that the role of pharmacists in the community has changed before and after COVID-19?” A total of 51.8% (*n* = 74) of the community pharmacy pharmacists, 55.4% (*n* = 36) of the drug store pharmacists and 47.9% (*n* = 57) of the hospital pharmacists reported that the role had changed (a little change or a big change) (Table 4). We also asked “Do you believe that pharmacists need to play a role in the community other than patient or citizen education regarding infectious diseases and public health?” A total of 82.5% (*n* = 118) of the community pharmacy pharmacists, 83.1% (*n* = 54) of the drug store pharmacists and 87.4% (*n* = 104) of the hospital pharmacists reported that they did consider this role (a little or a lot) (Table 4). In addition, we asked “Did the level of inquiries by patients or citizens regarding infection disease or infection prevention increase after COVID-19?” More than 80% (*n* = 116 and *n* = 61) of the community pharmacy and drugstore pharmacists chose “Yes”, while only 67.2% (*n* = 80) of the hospital pharmacists chose “Yes” (*p* < 0.01, chi-square test) (Table 4). Moreover, to understand pharmacists’ competency, we asked “Can you give guidance to citizens or patients if they ask you for information about infectious disease or infection prevention, etc.?” Only 26.6% (*n* = 38) of the community pharmacy pharmacists, 33.8% (*n* = 22) of the drugstore pharmacists and 46.2% (*n* = 55) of the hospital pharmacists answered “Yes, I can”, while 73.4% (*n* = 105) of the community pharmacy pharmacists, 66.1% (*n* = 43) of the drugstore pharmacists and 53.8% (*n* = 64) of the hospital pharmacists chose “Maybe I can” or “I’m not sure (I forgot)” (*p* < 0.01, chi-square test) (Table 4).

### 3.3. Pharmacists’ Skills Related to Public Health (Infection Prevention Education or Information Provision) in the Community

To understand the pharmacists’ skills, we asked “What are the instructions that you are not confident about?” The instruction items about which pharmacists were not confident were hand disinfection with alcohol (20.3% (*n* = 29) of the community pharmacy pharmacists, 16.9% (*n* = 11) of the drug store pharmacists and 3.4% (*n* = 4 numbers) of the hospital pharmacists) (*p* < 0.01, chi-square test), disinfection for each infectious disease (51.7% (*n* = 74) of the community pharmacy pharmacists, 38.5% (*n* = 25 numbers) of the drug store pharmacists and 30.3% (*n* = 36) of the hospital pharmacists) (*p* < 0.01, chi-square test) and explanation of infection route (25.9% (*n* = 37) of the community pharmacy pharmacists, 23.1% (*n* = 15) of the drug store pharmacists and 17.6% (*n* = 21) of the hospital pharmacists) (Table 5). Finally, we asked “If a seminar on public health such as infectious diseases and infection prevention were offered, would you like to participate and study again?” In total, 90.9% (*n* = 130) of the community pharmacists, 76.9% (*n* = 50) of the drugstore pharmacists and 84.0% (*n* = 100) of the hospital pharmacists chose “Yes” (*p* < 0.05, chi-square test) (Table 6).

## 4. Discussion

After the spread of COVID-19, not only citizens but also medical professionals have become more interested in infection prevention and public health. In particular, Japanese pharmacists are currently unable to administer vaccinations, but discussions have begun on the possibility that pharmacists could play such a role in the future [17]. Thus, the role of Japanese pharmacists is changing.

These changes in the role of pharmacists are occurring not only in Japan but also in other countries [14,18]. In addition, our previous study found that most citizens in Japan have not yet received education on infection prevention (related to hand washing and hand disinfection), although a certain degree of infection prevention can be achieved based on the lifestyle and behaviour of Japanese people [13]. Therefore, in our previous study, it was clarified that there is a need for citizens to receive infection prevention education from medical staff to ensure the effects of infection prevention [13]. In this study, we investigated changes in the perceptions and roles of pharmacists. Our study clarified that Japanese pharmacists recognize that the roles of pharmacists in the community are changing and realize that it is necessary to gain knowledge that is either lacking or new.

While 93.9% (*n* = 307) of the surveyed pharmacists showed an increased awareness of infection prevention, before the spread of COVID-19, 67.2% (*n* = 80) of hospital pharmacists were more aware of infection prevention than were 51.7 % (*n* = 74) of pharmacy pharmacists and 47.7% (*n* = 31) of drugstore pharma-cists. This finding indicates that hospital pharmacists, who have more opportunities to interact with inpatients than community pharmacies and drugstore pharmacists, are more conscious of infection prevention. In particular, in the Japanese system, one of the requirements for certification as an infection control specialist pharmacist (Board Certified Infection Control Pharmacy Specialist or Board Certified Pharmacist in Infection Control) is to be a member of the Japan Hospital Pharmacist Association, and the target of this pharmacist certification is hospital pharmacists [19]. These results are consistent with the finding that the hospital pharmacists who were asked to select infection prevention guidance items about which they had little or no self-confidence made fewer selections than did community pharmacy pharmacists and drugstore pharmacists across all items. In fact, in this study, there were significantly fewer hospital pharmacists who were less confident about teaching alcohol disinfection methods and disease-specific infection prevention than the other types of pharmacists.

After the spread of COVID-19, the number of pharmacists who felt that the role of pharmacists in the community had changed increased, but the numbers of community pharmacy pharmacists and drugstore pharmacists were found to be slightly higher than that of hospital pharmacists. There were significantly more community pharmacy pharmacists (81.1%, *n* = 116) and drugstore pharmacists (93.8%, *n* = 61) than hospital pharmacists (67.2%, *n* = 80) who answered that the number of questions regarding infectious diseases and infection prevention was increasing, and one of these reasons was considered to be the pharmacists’ close relationship with the community [20]. The role of pharmacists in the community in Japan is similar to that in other countries [21,22]. Especially in the case of drugstore pharmacists and community pharmacists, there are many general citizens who use drugstores or pharmacies to purchase over-the-counter drugs, infection prevention goods, and other items, without going to the hospital. Therefore, the results suggest that these individuals may ask about infection prevention methods while making their purchases.

However, when asked about infectious diseases and infection prevention methods, only 26.6% (*n* = 38) of the community pharmacists, 33.8% (*n* = 22) of the drugstore pharmacists, and 46.2% (*n* = 55) of the hospital pharmacists were confident that they could provide guidance to patients and citizens. On the other hand, 90.9% (*n* = 130) of the community pharmacy pharmacists, 76.9% (*n* = 50) of the drugstore pharmacists, and 84.0% (*n* = 100) of the hospital pharmacists answered that they would like to engage in continued education due to their lack of knowledge, which highlights the pharmacists’ attitudes as professionals. Therefore, these results suggest that more than half of all pharmacists are not confident in their knowledge because the pharmacists did not actively perform such work before the spread of COVID-19. In fact, experience-based knowledge differences have also been reported in the UAE (United Arab Emirates), and how long pharmacists have been engaged in a task has been shown to affect their skills [22]. In the future, in Japan’s ageing society, awareness of the prevention of illness will continue to increase, and it is expected that the number of ordinary citizens who use community pharmacies and drug stores rather than going to hospitals will increase. Therefore, by responding to the needs of citizens and patients, the role of pharmacists in the community is expected to change and expand even more.

As a limitation of our study, we might not have been able to correctly reflect drug store pharmacists’ opinions, as we could not recruit 100 drugstore pharmacists for the study. Moreover, we might not have truly grasped the needs of citizens, as pharmacists have not played a role in public health in the community until now. However, grasping pharmacists’ opinions through this survey and sharing these results might change the role of pharmacists in the community. Understanding pharmacists’ recognition of their roles will be the first step in reviewing undergraduate or postgraduate education. We are reviewing undergraduate and postgraduate education now in Japan. After few years later, new curriculum is opened, and the role of pharmacists in public health in community will be increased with changing of their recognition.

## 5. Conclusions

During the spread of COVID-19, the role of pharmacists in the community has gradually changed. In particular, at present, Japanese pharmacists do not have prescription rights or vaccination authority, but discussions have begun on whether vaccination should be included in the role of pharmacists. In addition, as various information flows exist about COVID-19 and related vaccines, it is becoming more necessary for pharmacists as medical professionals to be able to promptly obtain correct information and disseminate it to society. Therefore, information on infectious diseases and vaccines, and the sharing of such information, have already been implemented in various corporate organizations such as the Japan Pharmaceutical Association. In addition, as part of universities’ responsibility for the lifelong education of pharmacists, it is necessary to conduct teaching seminars on infectious diseases, infection prevention methods and public health, and to actively disseminate information while training pharmacists to meet the needs of citizens. In undergraduate and postgraduate education, the creation of a curriculum with an eye on the future, and depending on the needs of society, also needs to be considered.

Due to various social changes, many people need to change their behaviour. In addition, in a society where swift responses and changes are required, for individuals to work as medical personnel, their ability to respond while remaining aware of the needs of society is required now more than ever.

## Figures and Tables

**Table 1:** Characteristic of respondents.

Characteristic of Respondents	Category	Number (%)
**Q1. Age**	20 s	8 (2.4%)
	30 s	73 (22.3%)
	40 s	121 (37.0%)
	50 s	89 (27.2%)
	60 or older	36 (11.0%)
**Q2. Workplace**	Community pharmacy	143 (43.7%)
	Drug store	65 (19.9%)
	Hospital	119 (36.4%)
**Q3. Pharmacist History**	Less than 1 year	1 (0.3%)
	2–5 years	9 (2.8%)
	6–10 years	31 (9.5%)
	11–15 years	56 (17.1%)
	More than 16 years	230 (70.3%)
**Q4. Undergraduate** **Education**	4-year programme	294 (89.9%)
	6-year programme	33 (10.1%)
**Q5. Did Your Awareness of Infection Prevention Increase Due to the Impact of COVID-19?**	Yes	307 (93.9%)
	No	20 (6.1%)
**Q6. Since When Were Your Infection Prevention Measures Adopted?**	Every day from before the spread of COVID-19	119 (36.4%)
	Sometimes from before the spread of COVID-19	78 (23.9%)
	Every day after the spread of COVID-19	24 (7.3%)
	Sometimes after the spread of COVID-19	105 (32.1%)
	I am still not using such measures very much	1 (0.3%)

**Table 2:** Recognition of role in infection prevention education of citizens or patients.

Q7. Did You or Do You Have Recognition of Infection Prevention Education, etc., for Citizens or Patients as a Pharmacist?	“Yes” before the Spread of the COVID-19, *n* = 327	*p*-Value	“Yes” after the Spread of the COVID-19, *n* = 327	*p*-Value
Community pharmacist (*n* = 143)	74 (51.7%)	0.01 *	135 (94.4%)	0.02 *
Drugstore pharmacist (*n* = 65)	31 (47.7%)		63 (96.9%)	
Hospital pharmacist (*n* = 119)	80 (67.2%)		103 (86.6%)	

* *p* < 0.05, chi-square test.

**Table 3:** Conducted education about infectious diseases or prevention of infections.

Q8. What Did You or Do You Do to Educate Citizens Regarding Infectious Diseases or the Prevention of Infection?	Before the Spread of the COVID-19 *n* = 300			*p*-Value	After the Spread of the COVID-19 *n* = 300			*p*-Value
	Comm P	Drug P	Hosp P		Comm P	Drug P	Hosp P	
Gargling	39 (27.3%)	15 (23.1%)	33 (27.7%)	0.77	60 (42.0%)	28 (43.1%)	41 (34.5%)	0.37
Hand washing	48 (33.6%)	20 (30.8%)	50 (42.0%)	0.22	105 (73.4%)	42 (64.6%)	82 (68.9%)	0.41
Wearing masks when going out	29 (20.3%)	9 (13.8%)	20 (16.8%)	0.50	96 (67.1%)	43 (66.2%)	85 (71.4%)	0.68
Hand disinfection with alcohol	22 (15.4%)	9 (13.8%)	34 (28.6%)	0.01 *	101 (70.6%)	45 (69.2%)	79 (66.4%)	0.76
Disinfection for each infectious disease	4 (2.8%)	5 (7.7%)	13 (10.9%)	0.03 *	24 (16.8%)	21 (32.3%)	21 (17.6%)	0.02 *
Infection avoidance behaviour	9 (6.3%)	3 (4.6%)	7 (5.8%)	0.89	53 (37.1%)	24 (36.9%)	61 (51.3%)	0. 04 *
Explanation of infection route	15 (10.5%)	8 (12.3%)	22 (18.5%)	0.16	51 (35.7%)	26 (40.0%)	48 (40.3%)	0.70
Other	0		0.0		5		1.7	-

* *p* < 0.05, chi-square test. ▪ Comm P: Community pharmacy pharmacist; Drug P: Drug store pharmacist; Hosp P: Hospital pharmacist.

**Table 4:** Change of pharmacists’ role in the community.

**Q9. Do You Feel That the Role of Pharmacists in the Community Has Changed before and after the COVID-19? *n* = 327**				***p*-value**
Items	Comm P	Drug P	Hosp P	
No change	2 (1.4%)	3 (4.6%)	6 (5.0%)	0.82
Not much change	44 (30.8%)	18 (27.7%)	37 (31.1%)	
The same	23 (16.1%)	8 (12.3%)	19 (16.0%)	
A little change	61 (42.7%)	31 (47.7%)	43 (36.1%)	
Big change	13 (9.1%)	5 (7.7%)	14 (11.8%)	
Others	2 (1.4%)	3 (4.6%)	6 (5.0%)	
**Q10. Do You Consider That Pharmacists Need to Play the Role of Pharmacist in the Community Other than Patient or Citizens Education Regarding Infectious Diseases and Public Health? *n* = 327**				***p*-value**
Items	Comm P	Drug P	Hosp P	
Do not consider	0 (0.0%)	1 (1.5%)	1 (0.6%)	0.13
Do not much consider	25 (17.5%)	10 (15.4%)	14 (11.8%)	
Consider a little	89 (62.2%)	38 (58.5%)	67 (56.3%)	
Consider a lot	29 (20.3%)	16 (24.6%)	37 (31.1%)	
**Q11. Was the Level of Inquiries Increased by Patients or Citizens Regarding Infection Disease or Infection Prevention after COVID-19? *n* = 327**				***p*-value**
Items	Comm P	Drug P	Hosp P	
Yes	116 (81.1%)	61 (93.8%)	80 (67.2%)	0.00 **
No	22 (15.4%)	3 (4.6%)	34 (28.6%)	
Never before or now	5 (3.5%)	1 (1.5%)	5 (4.2%)	
**Q12. Can You Give Guidance to Citizens or Patients if They Ask You for Such Regarding Infectious Disease or Infection Prevention? *n* = 327**				***p*-value**
Items	Comm P	Drug P	Hosp P	
Yes I can	38 (26.6%)	22 (33.8%)	55 (46.2%)	0.00 **
Maybe I can (a little)	98 (68.5%)	37 (56.9%)	60 (50.4%)	
I’m not sure (I forgot)	7 (4.9%)	6 (9.2%)	4 (3.4%)	

** *p <* 0.01. ▪ Q9, 10: Kruskal-Wallis test; Q11, 12: Chi-square test. ▪ Comm P: Community pharmacy pharmacist; Drug P: Drug store Pharmacist; Hosp P: Hospital Pharmacist.

**Table 5:** Q13. What are the instructions that you are not confident about?

Not Confident Items	Comm P	Drug P	Hosp P	*p*-Value
Gargling	15 (10.5%)	7 (10.8%)	9 (7.6%)	0.67
Hand washing	19 (13.3%)	11 (16.9%)	8 (6.7%)	0.08
Wearing masks when going out	26 (18.2%)	9 (13.8%)	10 (8.4%)	0.07
Hand disinfection with alcohol	29 (20.3%)	11 (16.9%)	4 (3.4%)	0.00 **
Disinfection of each infectious disease	74 (51.7%)	25 (38.5%)	36 (30.3%)	0.00 **
Infection avoidance behaviour	22 (15.4%)	4 (6.2%)	12 (10.1%)	0.13
Explanation of infection route	37 (25.9%)	15 (23.1%)	21 (17.6%)	0.28
Others	0		0.0	-

** *p <* 0.01, chi-square test. ▪ Comm P: Community pharmacy pharmacist; Drug P: Drug store pharmacist; Hosp P: Hospital pharmacist.

**Table 6:** Pharmacists’ motivation to study again.

Q14 If a Seminar on Public Health Such as Infectious Diseases and Infection Prevention Were Offered, Would You Like to Participate and Study Again?	“Yes”	*p*-Value
Community pharmacist (*n* = 143)	130 (90.9%)	0.02 *
Drugstore pharmacist (*n* = 65)	50 (76.9%)	
Hospital pharmacist (*n* = 119)	100 (84.0%)	

* *p <* 0.05, chi-square test.

## Data Availability

The data can be found in this paper.
